# Cardiovascular Risk Factors and Subclinical Atherosclerosis in Greek Adolescents with Polycystic Ovary Syndrome: Its Relationship with Body Mass Index

**DOI:** 10.3390/children9010004

**Published:** 2021-12-22

**Authors:** Anastasia Garoufi, Athanasia Pagoni, Maria Papadaki, Antonios Marmarinos, Georgios Karapostolakis, Lina Michala, Alexandra Soldatou

**Affiliations:** 1 Lipid Outpatient Unit, 2nd Department of Pediatrics, Medical School, “P. & A. Kyriakou” Children’s Hospital, National and Kapodistrian University of Athens (NKUA), Thivon & Levadias Str., Goudi, 11527 Athens, Greece; garoufi@yahoo.gr (A.G.); nancy.pagoni@gmail.com (A.P.); papadaki.mairh@gmail.com (M.P.); 2 Laboratory of Clinical Biochemistry—Molecular Diagnostic, 2nd Department of Pediatrics, Medical School, NKUA, “P. & A. Kyriakou” Children’s Hospital, 11527 Athens, Greece; antonios_marmarinos@hotmail.com; 3 Radiology Department, 401 Military General Hospital of Athens (MGHA), 138 Mesogeion Av., 11525 Athens, Greece; georgioskarapostolakis@gmail.com; 4 First Department of Obstetrics and Gynaecology, Medical School, Alexandra General Hospital, National and Kapodistrian University of Athens (NKUA), 80 Vassilissis Sofias Av., 11528 Athens, Greece; linamichala@med.uoa.gr

**Keywords:** abdominal obesity, insulin resistance, lipid profile, small dense low-density lipoprotein cholesterol, visceral adiposity index, lipid accumulation product, carotid intima–media thickness, non-alcoholic fatty liver disease

## Abstract

Polycystic ovary syndrome (PCOS) is the most common endocrine condition affecting 6–18% of adolescents and is strongly associated with obesity and cardiovascular risk factors, enhancing the risk of atherosclerosis. Thirty-two adolescents with newly diagnosed PCOS were evaluated for lipid profile disorders, insulin resistance, inflammation, non-alcoholic fatty liver disease (NAFLD), and subclinical atherosclerosis through measurements of carotid intima–media thickness (cIMT). The relationships of the above markers with increased body mass index and abdominal obesity were investigated. Twenty-three adolescents (72%) were overweight (OW) or obese (OB). The OW/OB group had significantly higher insulin, HOMA-IR, high-sensitive C-reactive protein (hsCRP), visceral adiposity index (VAI), and lipid accumulation product (LAP) levels; and lower glucose-per-insulin ratios and HDL-C levels compared to the healthy weight group. The cIMT and small dense low-density lipoprotein cholesterol (sdLDL-C) levels did not differ between the two groups. Similarly, cIMT and sdLDL-C levels did not differ between PCOS-adolescents and healthy controls. CIMT was positively correlated with systolic blood pressure and waist circumference per height ratio. In conclusion, OW/OB PCOS-adolescents have a cluster of adverse factors predisposing them to atherosclerotic cardiovascular disease. Therefore, early cardiovascular risk assessment, as well as timely and targeted interventions, are necessary for prevention.

## 1. Introduction

Polycystic ovary syndrome (PCOS) is the most common endocrine disorder of women at reproductive age. Although evidence-based diagnostic criteria and management plans are well-established in adults, PCOS in adolescence presents several unresolved challenges [1,2]. The significant overlap of PCOS features with normal puberty hinders a widespread consensus on diagnostic criteria [3]. However, a recent metanalysis calculated the prevalence rates of adolescent PCOS based on three sets of criteria (Rotterdam, NIH, or AEPCOS) as 11%, 3.4%, and 8%, respectively [4]. Therefore, clinicians serving this age group must appropriately recognize PCOS, record important clinical features, and assess the presence of serious comorbidities.

Although there are studies that highlight important aspects in the evaluation of adolescents with PCOS [5], strategies for risk stratification of comorbidities remain unclear. Metabolic dysregulation observed in adolescents with PCOS presumably leads to long-term increased cardiovascular risk (CVR) [6,7]. In addition, although obesity is thought to worsen metabolic comorbidities [8], the extent of this association has not been clarified. There are limited clinical data available; therefore, the assessment of CVR and screening practices for comorbidities are controversial [9].

To this effect, the primary aim of our study was to investigate the association between CVR factors and increased body mass index in a cohort of adolescents with PCOS. A secondary aim was to examine whether increased c-IMT and/or sdLDL-C levels are correlated with PCOS, or other clinical and biochemical characteristics of adolescents associated with increased risk for cardiovascular disease.

## 2. Material and Methods

### 2.1. Study Population

Thirty-two menstruating adolescents aged 12.4 to 18.4 years (median age: 15.7 years) diagnosed with PCOS (hereafter, PCOS-adolescents) in the Outpatient Pediatric and Adolescent Gynaecology Clinic of the 1st Dept of Obstetrics and Gynaecology of the National and Kapodistrian University of Athens (NKUA), Alexandra General Hospital, were recruited in the study. Cardiovascular clinical, laboratory, and imaging markers were assessed at the Lipid Outpatient Unit of the 2nd Dept of Pediatrics of the NKUA, “P. & A. Kyriakou” Children’s Hospital. Healthy age-matched adolescents were recruited from the same unit. Inclusion criteria were Caucasian origin, menstruating adolescents, Tanner stage IV–V. Adolescents suffering from chronic diseases or taking any medications (including contraceptives) were excluded from the study. In addition, blood sampling was avoided during or near acute infectious diseases. The study was conducted in accordance with the Declaration of Helsinki and was approved by the Ethics Committee of the “P. & A. Kyriakou” Children’s Hospital (ethic code: 399/15-10-2012). Written informed consent was obtained from all participants and their parents prior to enrolment.

### 2.2. Diagnosis of PCOS

All participants had been diagnosed with PCOS, provided they fulfilled at least two out of three of the Rotterdam ESHRE/ASRM revised 2003 consensus criteria [10]. Clinical hyperandrogenism was assessed through the presence of hirsutism, determined as a Ferriman–Gallwey score (FGS) equal to or higher than 8 [11]. The increased levels on the third to fifth day of the adolescent’s menstrual cycle of either free or total testosterone, dehydroepiandrosterone sulfate (DHEAS), or androstenedione were used to define biochemical hyperandrogenism [10]. A pelvic ultrasound was performed during the follicular phase, to assess polycystic ovary morphology (PCOM) [10]. Furthermore, other disorders with similar clinical presentation, such as congenital adrenal hyperplasia including non-classic adrenal hyperplasia, Cushing’s syndrome and androgen-secreting tumors, were excluded for all participants.

### 2.3. Clinical Data Collection

A past medical history and a detailed physical examination were carried out. Body weight (BW) and height (Ht) were measured barefoot and lightly dressed, to the nearest 0.1 kg and 0.1 cm, respectively (TANITA, Corporation Tokyo). The body mass index (BMI) was calculated using the formula: BW in kg per Ht in m^2^. BMI classes were defined through the International Obesity Task Force (IOTF) criteria [12]. BW, Ht, and BMI z-scores and percentiles were also calculated with a standardized sex- and age-specific reference (CDC Growth calculator for 2 to 20 years: https://peditools.org/growthpedi/index.php accessed 30 August 2021). Waist circumference (WC) was measured to the nearest 0.1 cm, using a non-stretch measuring tape, with the individual in the standing position, naked at the abdomen; at midway point between the lower rib margin and upper edge of the iliac crest, at the end of normal expiration. The ratio of WC in cm to Ht in cm (WC/Ht) was calculated. Abdominal obesity was defined as a value of WC and WC/Ht ratio equal to or above the 90th age- and gender-specific reference percentiles for Greek adolescents [13]. Systolic (SBP) and diastolic (DBP) resting blood pressure was measured three timers with adolescents in a sitting position, using an automated oscillometric device (Dinamap V100, GE Medical Systems Information Technologies). The average value of three consecutive measurements was used for statistical analysis. All measurements were made by the same clinician.

### 2.4. Laboratory Data Collection

Venous 12 h fasting blood samples were obtained between 8 and 9 a.m. and used for determination of the lipid profile, sdLDL-C, glucose, insulin, hsCRP, alanine aminotransferase (ALT), aspartate aminotransferase (AST), gamma glutamyltranspeptidase (γGT), uric acid, creatinine, thyroid-stimulating hormone (TSH), and whole blood count. Serum total cholesterol (TC), high-density lipoprotein cholesterol (HDL-C), and triglycerides (TG) were measured with an enzymatic method (Roche Diagnostics, Cobass Integra 800 analyzer). Apolipoprotein A1 (apoA1) and apolipoprotein B (ApoB) were measured using an immunonephelometric assay (Siemens BNII Nephelometer Analyser). Lipoprotein (a), Lp(a), concentration was measured with N Latex Lp(a) Reagent with an immunonephelometric method, using a Behring nephelometer (Dade Behring, USA). Non-high-density lipoprotein cholesterol (Non-HDL-C) was calculated as TC minus HDL-C. Plasma low-density lipoprotein cholesterol (LDL-C) levels were directly measured using a homogenous enzymatic colorimetric assay (Roche Diagnostics, Cobas Integra 800 analyzer). All values are expressed in mg/dL. For the classification of lipid, lipoprotein, and apolipoprotein levels as “acceptable,” “borderline,” and “high” or “low,” the cutoff points recommended by the National Cholesterol Education Program Expert Panel on blood cholesterol levels in children and adolescents were used [14]. The atherogenic indexes LDL-C/HDL-C, LDL-C/ApoB, and TG/HDL-C were calculated.

The sdLDL-C levels were investigated in EDTA plasma isolated through centrifugation at 2000× *g* for 10 min at 4 °C. Plasma was placed in Eppendorf tubes and stored at −80 °C until further analysis. A direct quantitative determination of sdLDL-C (Roche Diagnostics, Cobas Integra 800, Roche Diagnostics GmbH, 68298 Mannheim, Germany) using a commercially available kit (sLDL-EX “SEIKEN”, Randox Laboratories Ltd., CV32TX Coventry, U.K.) was performed. Large buoyant low-density lipoprotein cholesterol (lbLDL-C) levels were calculated by subtracting sdLDL-C from the total LDL-C. The values are expressed in mg/dL. The absolute values of sdLDL-C and lbLDL-C, as well as the relative percentages of sdLDL-C on total LDL-C, were used in statistical analyses.

Lipid accumulation product (LAP) was calculated through the women-specific equation: [WC (cm) − 58] × TG (mmol/L) [15]. The visceral adiposity index (VAI) was estimated using the equation: WC (cm)/[36.58 + (1.89 × BMI)] × [TG (mmol/L)/0.81] × [1.52/HDL-C (mmol/L)] [16].

Serum fasting insulin and thyroid-stimulating hormone (TSH) levels were determined by an electrochemiluminescence immunoassay method (Roche Diagnostics GmbH, Mannheim, Germany) and expressed in µIU/mL. The homeostatic model assessment of insulin resistance (HOMA-IR) calculated by the formula: fasting glucose (mg/dL) × insulin (μU/mL)/405 and the glucose (mg/dL) to insulin (μU/mL) ratio were used to estimate insulin resistance (IR). hsCRP was measured using an immunonephelometric assay (Siemens BNII Nephelometer Analyser). A level of <1 mg/L was considered as desirable, whereas levels of 1 to 2.9 mg/L and ≥3 mg/L were considered as indicative of average and high risk.

All other parameters were determined using standard laboratory methods, according to the manufacturers’ instructions.

### 2.5. Imaging Studies Data Collection

In all participants, subclinical atherosclerosis was evaluated by measuring the intima–media thickness (IMT) of the right and left common carotid artery (cIMT) in mm by an expert radiologist using a B-mode ultrasound. The method used has been described in a previous study by our group [17]. The cIMT values were compared with those of age-matched healthy adolescents without PCOS, and their association with anthropometric, biochemical, and hormonal markers was evaluated. The radiologist was blinded to all clinical and biochemical characteristics of the subjects.

Non-alcoholic fatty liver disease (NAFLD) was assessed by liver ultrasound, performed by an experienced radiologist, using a high-resolution B-mode ultrasound system (General Electric Logig 7 ultrasound system) equipped with a 3–5 MHz convex probe. All measurements were based on a single sagittal image of the liver parenchyma and right kidney cortex during maximal inspiration. The liver fatty infiltration was grouped in grades: 0 (absent), 1 (mild), 2 (moderate), and 3 (severe). Grade 0 was defined as normal liver echotexture; grade 1 as slight and a diffuse increase in fine parenchymal echoes with normal visualization of diaphragm and portal vein borders and diaphragm; and grade 3 as fine echoes with poor or no visualization of portal vein borders, diaphragm, and posterior portion of the right lobe.

### 2.6. Statistical Analysis

PCOS-adolescents were subdivided according to BMI and comparisons were performed between overweight/obese and healthy weight groups. CIMT was also compared with that of age-matched adolescent girls without PCOS or dyslipidemia and sdLDL-C levels to those of healthy children and adolescents with normal lipid profiles.

Qualitative data are presented as absolute and relative frequencies (%). Depending on the distribution (normal or not), quantitative data are presented with average values, standard deviations, medians, and intra-quartile ranges. Regularity was checked using the Shapiro–Wilk criterion, and graphically with the use of histograms. The correlations between data and PCOS, cIMT, and sdLDL-C percentages on total LDL-C were determined with the use of Mann–Whitney and Kruskal–Wallis criteria for qualitative factors with two or more categories, respectively, as well as with the Spearman’s rho correlation coefficient for quantitative factors. Multiple linear regression was used, in order to investigate how correlations were affected by various potential confounders. Results are presented in β regression coefficients and 95% confidence intervals (CIs). Statistical analysis was performed with the statistical package SPSS v25 and a probability value of *p* < 0.05 was considered statistically significant.

## 3. Results

### 3.1. BMI and Cardiovascular Risk Factors in PCOS-Adolescents

A total of 9 out of 32 PCOS-adolescents (28%) had a healthy weight (BMI < 85th percentile for age), and 23 (72%) were overweight (*n* = 6, 19%) or obese (*n* = 17, 53%) with BMI values ≥85th but <95th percentile and ≥95th percentile for age, respectively. The clinical, biochemical, and imaging variables of our population, as well as their correlation with increased BMI, are presented in Table 1.

Overweight and obese adolescents had significantly higher WC and WC/Height ratios (*p* < 0.001 for both variables), as well as HOMA-IR (*p* = 0.001), insulin (*p* < 0.001), and hsCRP levels (*p* = 0.022), and a significantly lower glucose-to-insulin ratio (*p* < 0.001) and HDL-C levels (*p* = 0.005) compared to healthy weight adolescents. In addition, the LAP and the VAI were significantly higher (*p* < 0.001 and *p* = 0.038, respectively) in adolescents with increased BMI (Table 1). No significant differences were observed in other parameters of lipid profile, including sdLDL-C levels and its percentage on LDL-C, glucose concentration, liver and renal markers, and cIMT values between the two groups (*p* > 0.05).

A total of 22 (69%) and 21 out of 32 (66%) PCOS-adolescents had HOMA-IR values ≥2.7 and fasting insulin levels ≥12.2 μUI/mL, respectively. In 16 out 21 adolescents, insulin levels exceeded 17 μUI/mL. Overweight/obesity was observed in 90% of adolescents with increased HOMA-IR, and in 91% of those with increased insulin levels. Severe increases in hsCRP levels were also found in obese and overweight adolescents compared with normal-weight adolescents (39% vs. 11%). None of the adolescents had elevated LDL-C or non-HDL-C levels, whereas five had borderline levels. Eight (seven obese) adolescents had HDL-C levels lower than 45 mg/dL, and nine adolescents (six obese, three normal weight) had increased TGs levels (≥90 mg/dL). Uric acid was slightly above the upper normal limits in four (three obese) adolescents. Three adolescents had LAP values (≥42.7) indicative of NAFLD, and five had increased VAI values (≥1.78). Finally, 7 out 32 PCOS-adolescents (22%) had NFLAD (5 mild and 2 moderate grade). NFLAD was more frequent in obese adolescents compared to healthy weight adolescents (26% vs. 11%).

### 3.2. Subclinical Atherosclerosis in PCOS-Adolescents

CIMT values of PCOS-adolescents were compared to those of 29 healthy age-matched female controls. One more adolescent was added in the PCOS-adolescents group (*n* = 33). Clinical, laboratory, and imaging characteristics of the total population are shown in Table 2.

The median (IQR) cIMT-R, cIMT-L, and cIMT-Avg values were 0.44 (0.42, 0.47), 0.45 (0.42, 0.48), and 0.45 (0.43, 0.47), respectively, in PCOS-adolescents, versus 0.44 (0.41, 0.47), 0.44 (0.42, 0.47), and 0.44 (0.43, 0.47), respectively, in sex- and age-matched healthy controls. Kruskal–Wallis analysis showed no correlation of right, left, and average cIMT with PCOS status (*p* = 0.745, *p* = 0.357 and *p* = 0.450, respectively). The correlations of cIMT values with CVR factors in the total population are shown in Table 3.

A statistically significant positive correlation was found between cIMT and SBP (*p* < 0.001, *p* = 0.026, and *p* = 0.001 for cIMT-R, cIMT-L, and cIMT-Avg, respectively), BMI z-score (*p* = 0.002 and *p* = 0.003 for cIMT-L and cIMT-Avg, respectively), WC/Height (*p* = 0.005 and *p* = 0.007 for cIMT-L and cIMT-Avg, respectively), and DBP (*p* = 0.045 for cIMT-L). Following multiple linear regression analysis, the correlation of cIMT-R and cIMT-Avg with SBP remained statistically significant [(β = 0.002 95%CI (0.001–0.002), *p* < 0.001 and β = 0.001 95%CI (0.000–0.002), *p* = 0.002, respectively], as well as the correlation between cIMT-L and BMI z-score [(β = 0.017 95%CI (0.007–0.028), *p* = 0.002].

### 3.3. The Distribution of More Atherogenic Particles of LDL-C (sdLDL-C) in PCOS-Adolescents

The sdLDL-C levels of 29 PCOS-adolescents were compared with those of 27 healthy non-dyslipidemic controls. Clinical and laboratory characteristics of total population are shown in Table 4.

The absolute sdLDL-C levels (mg/dL), its percentage on the total LDL-C, and the sdLDL-C/lbLDL-C ratio did not differ significantly between PCOS-adolescents and healthy controls (Mann–Whitney: *p* = 0.140, *p* = 0.519, and *p* = 0.336, respectively). With the exception of TG levels (borderline significance, *p* = 0.051), no other significant correlation was found between the percentage (%) of sdLDL on total LDL-C levels and other clinical or biochemical CVR factors (Table 5).

## 4. Discussion

In the present study, the prevalence of certain CVR factors (CVRFs) was higher in overweight and obese PCOS-adolescents as compared with normal-weight adolescents. Hyperinsulinemia, insulin resistance, a more atherogenic lipid profile, chronic inflammation and NAFLD were observed. Compared to normal-weight PCOS-adolescents, overweight and obese adolescents had higher WC and WC/Ht ratio measurements; higher VAI, LAP, insulin, HOMA-IR, hsCRP, and TGs levels; and lower glucose-to-insulin ratios and HDL-C levels. In contrast, cIMT values did not differ between PCOS-adolescents with overweight/obesity and those with normal body weight, as well as between PCOS-adolescents and sex- and age-matched healthy controls, showing no indication of subclinical atherosclerosis among PCOS-adolescents. Notably, SBP levels had a significant impact on cIMT values. Similarly to cIMT, plasma concentrations of sdLDL-C did not differ significantly between PCOS-adolescents with increased and healthy weight, as well as between PCOS-adolescents and healthy controls. Finally, NAFDL was observed more frequently among overweight and obese PCOS-adolescents.

The prevalence of increased body weight in our population of PCOS-adolescents was very high (72%), with 53% being obese. Epidemiological data support the close association between obesity and PCOS, because a large percentage of women with PCOS ranging from 38% to 88% are either overweight or obese [18]. CVRFs such as insulin resistance, chronic inflammation, and lipid disorders were more prevalent in overweight and obese PCOS-adolescents compared with healthy-weight PCOS-adolescents. Specifically, 90% of adolescents with increased HOMA-IR and 91% of those with increased insulin levels, indicative of insulin resistance, were obese or overweight. Moreover, severe increases in hsCRP levels were found in obese and overweight PCOS-adolescents compared with normal-weight adolescents (39% vs. 11%). Therefore, in our population, increased body weight rather than the PCOS itself seemed to be associated with the high prevalence of CVRFs.

Existing studies concerning CVRFs and subclinical atherosclerosis in young women and adolescents with PCOS have presented conflicting results. This may be attributed to differences in methodology as well as the phenotypes of PCOS and BMI status examined [19,20,21]. In addition, most studies have compared CVRFs among obese adolescents with and without PCOS, or obese PCOS-adolescents and healthy-weight controls. Adult women with PCOS are generally considered at increased risk of early systemic atherosclerosis [22], although data regarding cardiovascular disease (CVD) risks in PCOS-adolescents are limited. In fact, a twofold higher incidence of CVRFs has been reported in obese adolescents with PCOS compared to healthy controls [23]. In another study, obese PCOS-adolescents were reported to present with several CVRFs when compared with normal-weight non-PCOS-adolescents, indicating a pro-atherogenic state in PCOS and obesity [24]. In contrast, in the study by Patel et al., obese PCOS-adolescents had more atherogenic CVRFs than obese non-PCOS-adolescents [25]. Similarly, an increased prevalence of CVRFs including IR, dyslipidemia, and metabolic syndrome was noted in obese adolescents with PCOS compared to those with normal weight, as well as to their age- and BMI-matched non-PCOS counterparts [26]. Impaired glucose tolerance and/or IR have been shown in 39% of PCOS-adolescents [27]. In a recent study, 18.2% of PCOS-adolescents had HOMA-IR ≥2.7 and 35.7% had fasting insulin ≥12.2 µUI/mL: cut-off levels used to define insulin resistance [28].

Many studies of young women with PCOS support an independent association of PCOS with IR, with the susceptibility to increase further with increasing BMI [18,29]. Similarly to adult women, this association seems independent of BMI status, but worsened by concomitant obesity among adolescents [29]. On the other hand, hyperinsulinemia and IR play a crucial role in the pathogenesis of PCOS in women, and the severity of hyperinsulinemia has been correlated with the severity of clinical manifestations [30]. Among adolescents, the prevalence of IR is higher in those with the classic phenotypes of PCOS and exacerbated in those with overweight/obesity [29]. Indeed, although higher levels of fasting glucose, serum insulin, and HOMA-IR were found in both obese and normal-weight young women with PCOS compared to their BMI-matched controls, only obese women with PCOS had measurements above the reference range [31].

In our study, the only lipid disorder found was significantly lower HDL-C levels in overweight and obese PCOS-adolescents compared to normal-weight adolescents. Eight adolescents had HDL-C levels <45 mg/dL, seven of whom were obese. All other lipid-lipoprotein levels were similar between the two groups. Furthermore, none of the adolescents had high levels of LDL-C or non-HDL-C. TGs were increased (≥90 mg/dL) in six obese and three normal-weight adolescents. Lower HDL-C levels have been consistently reported in previous studies in both PCOS-adolescents and young women compared with obese and normal-weight non-PCOS controls [24,25,32]. Lower HDL-C levels have also been reported in obese PCOS-women compared with non-obese women [28]. In contrast, in the study by Vine et al., lipid levels were similar between adolescents with and without PCOS [23]. Several of the above-mentioned studies have also shown a more atherogenic lipid profile among PCOS patients [24,25,32].

In the present study, the sdLDL-C and lbLDL-C levels, as well as the percentage of sdLDL-C on total LDL-C, did not differ between PCOS-adolescents with increased and normal weight as well as between the total PCOS population and healthy controls. As in our study, no quantitative or qualitative differences in the LDL-C profile were found in normal-weight Korean women with PCOS, as well as in the analysis of hyperandrogenic and non-hyperandrogenic PCOS subgroups [33]. Higher sdLDL-C and lbLDL-C levels in PCOS-women than in healthy controls have also been reported, with the values being within the normal reference range [34]. In a cohort of U.S. women, adverse LDL patterns were attributed to the PCOS status [35].

Finally, Lp(a) levels did not differ in the two groups of PCOS-adolescents examined in our study. There are limited data regarding the evaluation of Lp(a)) measurements as a marker of CVD risk in women and adolescents with PCOS. Increased Lp(a) levels have been shown in one-third of PCOS-women, particularly those with hyperandrogenism. In addition, non-obese PCOS-women had higher Lp(a) concentrations compared to healthy controls [33].

In our study population, the lipid accumulation product (LAP) values were significantly higher in PCOS-adolescents with increased body weight compared with healthy-weight adolescents. The LAP, an index of excessive hepatic lipid accumulation, was first introduced by Kahn in adults to predict CVR [36]. As in adults, LAP values could be used to predict NAFLD in obese children and adolescents [37]. In a cohort of obese children, positive correlations of LAP included pubertal stage, fasting insulin, and HOMA-IR. Furthermore, NAFLD was associated with significantly higher LAP values [38].

We observed a higher visceral adiposity index (VAI) in the group of overweight/obese PCOS-adolescents compared to the healthy-weight group. In a study of Indian girls with PCOS, the mean VAI was significantly higher than in controls, and a one-unit increase in the VAI score was associated with a 5.23-fold higher risk of metabolic syndrome (MetS) [39]. The VAI was the best predictor of MetS in both obese and non-obese women with PCOS [28]. In addition, an increased risk of CVD based on the VAI has been reported in PCOS-adolescents with metabolic disorders and excess weight [27].

In the present study, hsCRP levels were significantly higher in obese PCOS-adolescents compared to normal-weight adolescents. In addition, severe elevated levels were found mainly in adolescents with increased body weight (33% vs. 11%). Increased markers of inflammation, including CRP, have previously been found in obese PCOS-adolescents compared with normal-weight non-PCOS-adolescents [24]. Obesity and IR are considered the main predictive factors of increased CRP [40]. In a recent study, CRP was increased in young women with PCOS, irrespective of obesity, compared to their BMI-adjusted controls, suggesting a baseline risk of low-grade inflammation and CVD among PCOS-women [31]. Obesity status was deemed important in a cohort of adolescent girls, because obese girls with PCOS without IR had higher levels of CRP compared to girls with normal BMI values with or without metabolic disorders. Therefore, excess weight, even in the absence of metabolic disorders, may trigger chronic inflammation in PCOS-adolescents [27].

We found that 22% of PCOS-adolescents had a liver ultrasound indicative of mild or moderate NAFLD, predominantly among those with obesity. In the general pediatric population, NAFLD is the most common cause of chronic liver disease, particularly among children with obesity and MetS [41]. Similarly to PCOS, the risk of NAFLD seems to increase in the face of excess adiposity and IR. In addition, PCOS-adolescents with NAFLD had greater adiposity and HOMA-IR than those with NAFLD without a PCOS diagnosis. The metabolic phenotype of adolescents with NAFLD and PCOS was similar to that of boys with NAFLD. Therefore, PCOS was highlighted as an independent predictor of NAFLD in adolescent girls [42].

We did not observe early subclinical atherosclerosis in PCOS-adolescents with increased or normal body weight, as assessed with the measurement of cIMT. In addition, cIMT values did not differ between PCOS-adolescents and age-matched healthy adolescent girls. The results of studies evaluating cIMT in adolescents and young women with PCOS are contradictory. Some studies have demonstrated higher cIMT values in those with PCOS compared to age- and/or BMI-matched non-PCOS controls, whereas others have shown no difference between PCOS women and controls, independently of obesity, and between obese and normal-weight women independently of PCOS [19,20,24,43,44]. In the study by Patel et al., obese adolescents with PCOS had higher cIMT measurements than obese adolescents without PCOS [25]. In addition, no differences were observed between women with different PCOS phenotypes [45]. The discrepant results on cIMT values between studies might be due to the different prevalence of PCOS phenotypes and cardiovascular risk factors, as well as in methodological differences between the studies regarding study populations and methods of cIMT measurement [19,21]. Furthermore, the shorter duration of hyperandrogenism, obesity, and co-morbidities among PCOS-adolescents compared to young women with PCOS may be implicated in the observed differences [19,24]. In addition to a significant positive association of increased CIMT with the duration of disease, the diagnosis of PCOS was the strongest predictor of CIMT, after adjustment for confounding factors, explaining 70% of its variability [20].

According to our study, only the BMI z-score and SBP values had a significant impact on cIMT measurements in adolescents with and without PCOS. Similarly, BMI has been found to be a predictor of cIMT in young women with PCOS [20]. Moreover, a positive correlation between cIMT and SBP has also been observed in other studies [20]. We did not find any significant correlations between cIMT and hsCRP, lipid profile, and markers of insulin resistance. These results are consistent with those of a previous study which showed no correlation between cIMT and hsCRP, although hsCRP was greater in obese PCOS-adolescents compared with normal-weight non-PCOS-adolescents [24]. In terms of lipid profile disorders and insulin resistance, some studies in young women with PCOS have shown a positive correlation of cIMT with atherogenic lipoproteins such as LDL-C and triglycerides, and with IR, and a negative correlation with the anti-atherogenic HDL-C [20,46]. The fact that we did not find a correlation between PCOS status and some of the CVR factors or cIMT may be, in part, due to the small number of participants in the study.

## 5. Limitations and Strengths of the Study

The main limitation of our study was the small number of observations, particularly in the non-obese group, which did not allow us to subdivide adolescents according to PCOS phenotype and to perform more comparisons and correlations. The number of adolescents recruited was determined by the number of adolescents with newly diagnosed PCOS prior to the initiation of any treatment identified, and not a formal power calculation. Another limitation is the lack of the same number of age-matched non-PCOS-adolescents with increased and normal weight, respective to the number of PCOS-adolescents, as controls. However, there are at least two strengths of the present study to mention. First, all comparisons concerned normal-weight and excess-weight PCOS-adolescents. Secondly, cIMT values of PCOS-adolescents were compared with those of age-matched non-PCOS and non-dyslipidemic adolescents.

## 6. Conclusions

Our findings suggest that obese PCOS-adolescents present with a cluster of CVRFs predisposing them to early atherosclerosis. We observed that the greatest impact of CVRFs on obese PCOS-adolescents may be due to obesity and not to PCOS itself. However, a larger number of Greek PCOS-adolescents, particularly those with normal weight, and a non-PCOS age- and BMI-matched control group, are necessary to confirm and potentially strengthen these findings. Despite the identification of CVRFs, the lack of subclinical atherosclerosis in our cohort is significant for identifying a window of opportunity to avoid the long-term risk of atherosclerotic cardiovascular disease. Indeed, our results in adolescents with PCOS, especially in those with increased body weight, are promising. The early identification of CVRFs, followed by timely interventions, mainly with lifestyle modification and weight loss, as well as a tighter follow-up, are needed to change the lifetime course of adolescents with PCOS for whom we provide care.

## Figures and Tables

**Table 1:** Correlation of clinical, biochemical, and imaging variables with increased BMI (Mann–Whitney).

	Normal Weight (*n* = 9)	Overweight and Obese (*n* = 23)	
	Median, IQR	Median, IQR	*p*-Value
BW z-score	0.34 (−0.43–0.69)	1.78 (1.41–2.14)	<0.001
Height z-score	−0.05 (−0.55–0.40)	0.14 (−0.46–1.03)	0.386
BMI z-score	0.44 (−0.78–0.71)	1.81 (1.58–2.10)	<0.001
WC (cm)	71.00 (62.00–78.50)	89.00 (84.00–93.00)	<0.001
WC/Height	0.44 (0.37–0.48)	0.55 (0.51–0.57)	<0.001
Age (years)	16.20 (15.40–17.00)	15.10 (14.30–16.60)	0.281
SBP (mm Hg)	114.00 (104.50–121.50)	121.00 (109.00–129.00)	0.094
DBP (mm Hg)	65.00 (62.50–69.00)	72.00 (64.00–79.00)	0.213
cIMT-R (mm)	0.43 (0.41–0.46)	0.45 (0.42–0.47)	0.321
cIMT-L (mm)	0.44 (0.42–0.46)	0.48 (0.43–0.49)	0.229
cIMT-Avg (mm)	0.44 (0.42–0.44)	0.46 (0.43–0.48)	0.122
TC (mg/dL)	167.00 (148.00–184.50)	148.00 (140.00–158.00)	0.064
LDL-C (mg/dL)	91.00 (69.40–103.90)	79.00 (70.60–97.80)	0.386
HDL-C (mg/dL)	65.00 (54.00–74.50)	48.00 (42.00–58.00)	0.005
Non-HDL-C(mg/dL)	102.00 (80.00–120.50)	98.00 (85.00–117.00)	0.621
TGs (mg/dL)	61.00 (45.00–93.50)	67.00 (52.00–92.00)	0.321
sdLDL-C (mg/dL)	23.40 (18.05–28.65)	17.40 (14.60–25.17)	0.216
sdLDL-C (%LDL-C)	23.60 (22.28–31.70)	22.54 (17.20–27.21)	0.234
lbLDL-C (mg/dL)	64.60 (53.60–84.55)	67.25 (57.80–71.27)	0.908
lbLDL-C (%LDL-C)	76.39 (68.29–77.71)	77.45 (72.78–82.79)	0.234
SdLDL/lbLDL ratio	0.30 (0.28–0.46)	0.29 (0.20–0.37)	0.234
hsCRP (mg/L)	0.38 (0.16–1.69)	1.34 (0.64–4.55)	0.022
Glucose (mg/dL)	87.00 (84.00–92.50)	84.00 (78.00–90.00)	0.213
Insulin (IU/L)	8.83 (7.80–11.65)	21.44 (13.01–31.12)	<0.001
Gl/Ins ratio	9.97 (7.49–11.38)	3.59 (2.60–6.69)	<0.001
HOMA-IR	1.91 (1.59–2.53)	4.10 (2.87–6.22)	0.001
AI-1 (LDL-C/HDL-C)	1.32 (1.06–1.82)	1.73 (1.32–2.29)	0.122
AI-2 (LDL-C/ApoB)	1.35 (1.27–1.41)	1.32 (1.28–1.38)	0.681
AI-3 (TG/HDL-C)	0.95 (0.66–1.68)	1.45 (1.10–2.00)	0.058
Uric acid (mg/dL)	4.00 (3.35–5.30)	5.10 (4.40–5.50)	0.094
ALT (IU/L)	14.00 (12.50–16.50)	16.00 (13.00–20.00)	0.386
AST (IU/L)	12.00 (11.00–17.00)	16.00 (12.00–36.00)	0.133
γ-GT (IU/L)	10.00 (9.00–16.00)	13.50 (11.00–19.00)	0.086
Cr (mg/dL)	0.70 (0.60–0.90)	0.80 (0.70–0.85)	0.499
VAI	0.68 (0.51–1.20)	1.04 (0.80–1.52)	0.038
LAP	8.80 (2.85–13.65)	25.00 (17.40–33.40)	<0.001
FGS	3.50 (1.00–10.75)	7.00 (2.00–10.00)	0.642

BW: body weight; BMI; body mass index; WC: waist circumference; SBP: systolic blood pressure; DBP: diastolic blood pressure; cIMT: carotid intima–media thickness; TC: total cholesterol; LDL-C: low-density lipoprotein cholesterol; HDL-C: high-density lipoprotein cholesterol; Non-HDL-C: non-high-density lipoprotein cholesterol; TGs: triglycerides; sdLDL-C: small dense low-density lipoprotein cholesterol; lbLDL-C: large buoyant low-density lipoprotein cholesterol; hsCRP: high-sensitivity C-reactive protein; Gl/Ins: glucose/insulin; HOMA-IR: homeostatic model assessment of insulin resistance; AI: atherogenic index; ALT: alanine aminotransferase; AST: aspartate aminotransferase; γGT: gamma glutamyltranspeptidase; Cr: creatinine; VAI: visceral adiposity index; LAP: lipid accumulation product; FGS: Ferriman–Gallwey score.

**Table 2:** Population clinical, laboratory, and imaging data.

	Ν = 62 (100%)
Body Mass Index	
Normal	30 (48.4%)
Overweight	10 (16.1%)
Obese	22 (35.5%)
	Μean (SD)
cIMT-R (mm)	0.45 (0.04)
cIMT-L (mm)	0.45 (0.04)
cIMT-Avg (mm)	0.45 (0.03)
SBP (mmHg)	114.73 (12.27)
DBP (mmHg)	66.37 (8.33)
LDL-C (mg/dL)	90.74 (21.82)
Non-HDL-C (mg/dL)	103.35 (23.18)
	Median (IQR)
Age (years)	15.00 (14.00–16.45)
WC/Height ratio	0.49 (0.42–0.55)
BMI Z-score	1.09 (0.21–1.71)
TGs (mg/dL)	64.50 (51.75–87.25)
Lp(a) (mg/dL)	9.45 (5.00–18.05)

cIMT: carotid intima–media thickness; SBP: systolic blood pressure; DBP: diastolic blood pressure; LDL-C: low-density lipoprotein cholesterol; Non-HDL-C: non-high-density lipoprotein cholesterol; WC: waist circumference; BMI; body mass index; TGs: triglycerides; Lp(a): lipoprotein (a).

**Table 3:** Correlations of cIMT with clinical or laboratory quantitative parameters (Spearman’s rho).

	*n*	Rho	*p*-Value	Rho	*p*-Value	Rho	*p*-Value
Age (years)	62	0.020	0.880	0.169	0.188	0.133	0.304
BMI z-score	62	0.214	0.095	0.393	0.002	0.372	0.003
WC/Height ratio	59	0.221	0.093	0.362	0.005	0.347	0.007
SBP (mmHg)	62	0.444	<0.001	0.282	0.026	0.402	0.001
DBP (mmHg)	62	0.080	0.534	0.253	0.045	0.186	0.144
LDL-C (mg/dL)	62	0.213	0.096	0.069	0.596	0.154	0.231
Non-HDL-C(mg/dL)	62	0.161	0.213	0.091	0.481	0.139	0.283
TGs (mg/dL)	62	0.008	0.950	0.229	0.074	0.142	0.269
Lp(a) (mg/dL)	50	0.097	0.502	0.131	0.366	0.152	0.292

cIMT: carotid intima–media thickness; SBP: systolic blood pressure; DBP: diastolic blood pressure; LDL-C: low-density lipoprotein cholesterol; Non-HDL-C: non-high-density lipoprotein cholesterol; WC: waist circumference; BMI; body mass index; TGs: triglycerides; Lp(a): lipoprotein (a).

**Table 4:** Population clinical and laboratory data.

	N = 56 (100%)
	Mean (SD)
BW z-score	1.10 (0.89)
Height z-score	0.49 (0.97)
DBP (mmHg)	67.21 (7.96)
TC (mg/dL)	165.54 (24.55)
LDL-C (mg/dL)	95.91 (18.99)
Apo B (mg/dL)	71.36 (13.80)
AI-1 (LDL-C/HDL-C)	1.77 (0.59)
AI-2 (LDL-C/ApoB)	1.34 (0.13)
	Median (IQR)
BMI z-score	1.34 (0.36–1.74)
SBP (mmHg)	118.00 (107.25–124.50)
HDL-C(mg/dL)	54.00 (46.00–65.00)
Non-HDL-C (mg/dL)	113.50 (89.00–121.75)
TGs (mg/dL)	63.00 (47.25–83.75)
Apo A1 (mg/dL)	142.00 (130.50–155.75)
Al-3 (TG/HDL-C)	1.04 (0.76–1.84)
hsCRP (*n* = 45)	0.54 (0.26–2.01)

BW: body weight; DBP: diastolic blood pressure; TC: total cholesterol; LDL-C: low-density lipoprotein cholesterol; Apo B: apolipoprotein B; AI: atherogenic index; BMI; body mass index; WC: waist circumference; SBP: systolic blood pressure; HDL-C: high-density lipoprotein cholesterol; Non-HDL-C: non-high-density lipoprotein cholesterol; TGs: triglycerides; Apo A1: apolipoprotein A1; hsCRP: high-sensitivity C-reactive protein.

**Table 5:** Correlation of sdLDL-C % on total LDL-C with clinical and biochemical parameters (Spearman’s rho).

	sdLDL% on LDL-C		
	*n*	Rho	*p*-Value
BW z-score	56	−0.242	0.072
Height z-score	56	0.005	0.969
BMI z-score	56	−0.221	0.102
SBP (mmHg)	56	−0.162	0.232
DBP (mmHg)	56	−0.027	0.842
TC (mg/dL)	56	0.182	0.178
LDL-C (mg/dL)	56	−0.077	0.571
HDL-C (mg/dL)	56	0.230	0.089
Non-HDL-C (mg/dL)	56	0.034	0.082
TGs (mg/dL)	56	0.262	0.051
Apo A1 (mg/dL)	56	0.209	0.123
Apo B (mg/dL)	56	0.051	0.708
AI−1 (LDL-C/HDL-C)	56	−0.105	0.442
AI-2 (LDL-C/ApoB)	56	0.072	0.600
A1–3 (TG/HDL-C)	56	0.091	0.505
hs CRP (mg/L)	45	−0.269	0.074

BW: body weight; BMI; body mass index; SBP: systolic blood pressure; DBP: diastolic blood pressure; TC: total cholesterol; LDL-C: low-density lipoprotein cholesterol; HDL-C: high-density lipoprotein cholesterol; Non-HDL-C: non-high-density lipoprotein cholesterol; TGs: triglycerides; Apo A1: apolipoprotein A1; Apo B: apolipoprotein B; AI: atherogenic index; hsCRP: high-sensitivity C-reactive protein.
